# Deep learning for prediction of hepatocellular carcinoma recurrence after resection or liver transplantation: a discovery and validation study

**DOI:** 10.1007/s12072-022-10321-y

**Published:** 2022-03-29

**Authors:** Zhikun Liu, Yuanpeng Liu, Wenhui Zhang, Yuan Hong, Jinwen Meng, Jianguo Wang, Shusen Zheng, Xiao Xu

**Affiliations:** 1 Department of Hepatobiliary and Pancreatic Surgery, The Center for Integrated Oncology and Precision Medicine, Affiliated Hangzhou First People’s Hospital, Zhejiang University School of Medicine, 261 HuanSha Road, Hangzhou, 310006 China; 2 Department of Hepatobiliary and Pancreatic Surgery, The First Affiliated Hospital, Zhejiang University School of Medicine, 79 Qingchun Road, Hangzhou, 310003 China; 3 NHC Key Laboratory of Combined Multi-organ Transplantation, Hangzhou, 310003 China; 4 School of Mathematical Sciences, Zhejiang University, Hangzhou, 310058 China; 5 Department of Electrical Engineering and Computer Science, Syracuse University, 4-206 Center for Science and Technology, Syracuse, NY 13244-4100 USA

**Keywords:** Deep learning, MobileNetV2, HCC, Prognosis, LT

## Abstract

**Background:**

There is a growing need for new improved classifiers of prognosis in hepatocellular carcinoma (HCC) patients to stratify them effectively.

**Methods:**

A deep learning model was developed on a total of 1118 patients from 4 independent cohorts. A nucleus map set (*n* = 120) was used to train U-net to capture the nuclear architecture. The training set (*n* = 552) included HCC patients that had been treated by resection. The liver transplantation (LT) set (*n* = 144) contained patients with HCC that had been treated by LT. The train set and its nuclear architectural information extracted by U-net were used to train the MobileNet V2-based classifier (MobileNetV2_HCC_class). The classifier was then independently tested on the LT set and externally validated on the TCGA set (*n* = 302). The primary outcome was recurrence free survival (RFS).

**Results:**

The MobileNetV2_HCC_class was a strong predictor of RFS in both LT set and TCGA set. The classifier provided a hazard ratio of 3.44 (95% CI 2.01–5.87, *p* < 0.001) for high risk versus low risk in the LT set, and 2.55 (95% CI 1.64–3.99, *p* < 0.001) when known prognostic factors, remarkable in univariable analyses on the same cohort, were adjusted. The MobileNetV2_HCC_class maintained a relatively higher discriminatory power [time-dependent accuracy and area under curve (AUC)] than other factors after LT or resection in the independent validation set (LT and TCGA set). Net reclassification improvement (NRI) analysis indicated MobileNetV2_HCC_class exhibited better net benefits for the Stage_AJCC beyond other independent factors. A pathological review demonstrated that tumoral areas with the highest recurrence predictability featured the following features: the presence of stroma, a high degree of cytological atypia, nuclear hyperchromasia, and a lack of immune cell infiltration.

**Conclusion:**

A prognostic classifier for clinical purposes had been proposed based on the use of deep learning on histological slides from HCC patients. This classifier assists in refining the prognostic prediction of HCC patients and identifies patients who have been benefited from more intensive management.

**Supplementary Information:**

The online version contains supplementary material available at 10.1007/s12072-022-10321-y.

## Introduction

Hepatocellular carcinoma (HCC) is the seventh most common malignant tumor and the third primary cause of mortality worldwide [1, 2]. The prevalence of HCC is relatively high in the Asia–Pacific countries [3]. Hepatectomy and liver transplantation (LT) are still the main treatment approaches of HCC. Despite significant progress in diagnostic and management techniques for HCC, the recurrence rate is still high (up to 70–80%) following hepatectomy and 20–40% following LT [4–6]. A refinement of prognostic models, especially those based on the accessible data, could easily allow for detection of early warning signs during follow-up and prolonging adjuvant therapeutic decisions [7]. The establishment of precise HCC recurrence model for HCC patients after operation is difficult. Several clinical risk factors, using conventional Cox proportional hazards regression, have been identified to predict HCC recurrence, including maximum tumor diameter, tumor number, tumor differentiation, macrovascular invasion, serum alpha-fetoprotein (AFP) level [8–11]. Metroticket 2.0 Model was developed based on level of AFP, tumor size, and tumor number, to determine risk of death from HCC-related factors after liver transplantation using a competing-risk Cox_PH regression analysis [12]. In addition, the biomarkers such as genes, proteins, and miRNAs are investigated and integrated into the diagnosis and prognosis model [13–15]. miRNAs with the advantage of reliability are good choice for biomarkers. The biomarkers were proved to have a better survival prediction power than tumor-node-metastasis (TNM) stage (miRNA-AUC test = 0.64 vs. TNM-AUC test = 0.61) [16]. However, the biomarkers suffer from economic and time costs. The widely available pathology slides contain morphological markers of disease progression [17, 18], which is not routinely used to objectively extract prognostic biomarkers.

The prognosis of a disease is closely associated with its pathological features. Histological analysis of tumor tissues can certainly provide information for patient stratification and treatment allocation. Histological slides contain a vast amount of information that can be quantitatively assessed by deep learning algorithms. In recent times, convolutional neural networks (CNNs) have been extensively employed in the fields of speech recognition, traffic sign management, and face recognition [8–10]. CNNs have successfully passed numerous image interpretation tests and also retrieved extra information from histopathological images. Recently, a pioneering survey revealed that simulating routine pathology workflows, i.e., using subset algorithms based on deep learning, achieved a better diagnostic performance than an exert group consisting of 11 pathologists in monitoring lymph node metastasis in breast cancers [19]. Coudray et al. have shown in their work that CNNs successfully identified the prime histological subtypes of non-small cell lung cancer and predicted the gene mutation status of genes such as *STK11* and *EGFR* [20]. CNNs are also found to be useful for predicting the aggressiveness of colorectal cancer [21]. More recently, there is growing evidence that suggests the computational processing applied on histological slides better refines prediction for patient prognosis, thus leading to an improvement in treatment allocation. A model based on deep learning developed by Saillard et al. could correctly predict the survival in HCC patients. They used pre-trained CNNs to extract features from images and then the network selected 25 tiles with maximum and minimum scores to predict patient survival [22]. Skrede et al. successfully developed a marker to predict the prognosis of colorectal cancer in large cohorts using MobileNet V2, one of the CNNs [17], building the model using Multiple Instance Learning (MIL). Interestingly, the local spatial arrangement of nuclei in the histopathology images proved to be vital information of high prognostic value in cases of oropharyngeal cancer [23].

In the present study, four independent cohorts of postoperative HCC patients were investigated for developing and validating a MobileNet V2-based model to improve prognosis and prediction. The scientific and innovative features of our method are inspired by two studies: (1) the study by Skrede et al. trained MobileNet V2 using the MIL, which allowed training on large tile collections labeled with the associated whole-slide images [17], and (2) the study by Ji et al. in which nuclear architectural information was used in building a model, which proved to be effective for cancer grading and prediction of patient outcomes [24]. To capture localized nuclear architectural information in the independent cohort, local nuclei measurements were constructed by U-net, a convolutional networks for biomedical image segmentation [25]. Here we conclude that the models have higher accuracy in survival prediction relative to conventional methods. Our research aimed at using MobileNet V2 to analyze histopathology images and propose an automatic prognostic classifier exclusive to HCC patients that have undergone liver resection. In addition, we validated the prognostic power of MobileNet V2 across different cohorts.

## Methods

### Patients and samples

Four different cohorts were enrolled in this study. Stained tumor tissue sections obtained from patients with adequate quality and tiles were used. The first cohort was used to train U-net to capture the localized nuclear architectural information (Nucleus map set, *n* = 120). The second cohort was from HCC patients who had received surgical resection treatment from the First hospital of Zhejiang University in 2010–2016 and have a so-called distinct outcome (Train set, *n* = 552). Patients exhibiting obvious good or bad outcomes (good: 274, poor: 278) were used as training cohorts. Patients with a 4-year follow-up history after resection and no recorded recurrence were grouped under the good outcome cohort. At the same time, the bad outcome group included patients who relapsed within a period of 1.6 years (exclusive) after surgery. The third cohort was from HCC patients who received liver transplantation from the First hospital of Zhejiang University between 2015 and 2019 (LT set, *n* = 144). The nucleus map set, train set, and LT set were collated from these three different batches of HCC patients after obtaining approval from the ethics committee of the institution. A fourth dataset, namely the TCGA set, with complete follow-up data (*n* = 302) from the TCGA database, was included for external validation.

The nucleus map set, train set, and LT set were scanned and digitized using a P250FLASH2 (3DHISTECH3) at 20 × magnification. Nucleus map sets were used for training U-net. The train set was used to train the MobileNet V2, while the LT set was used to externally validate the model in HCC treated by LT. The histology slides, clinical follow-up data, and histological annotation were retrieved from the TCGA database (https://cancergenome.nih.gov/).

### Tile cropping and color normalization

Due to the limitations of graphic card memory, it was almost impossible to process whole-sliced pathological images, which are usually at a resolution of 100,000 × 100,000, on GPU or main memory during the training phase. To circumvent this problem, the current best practice is to cut large images into hundreds of smaller images, which are called tiles or patches [17, 26, 27]. In our study, these tiles were 512 × 512 pixels (px) and 0.25 µm per px. They were cropped from the nucleus map set, train set, and LT set. They were finally resized to a resolution of 224 × 224 px. The tiles were normalized as described previously [28].

### Extending features with a segmentation heat map of nuclear architectural information using U-net

Before feeding these data into the model, we used a trained image segmentation model to get the heat map of nuclei segmentation for each tile. The segmentation model was a U-net neural network trained with a nucleus map set. Let $I$ denote an image slice, $p$ indicate the U-net output, and $y$ denote the ground-truth label in the image slice where $\epsilon = 0.00000001$ is a smoothing term to make the denominator non-zero. The loss function is Dice loss (1) and the final Dice Score on the TCGA test set can reach up to 82%. The segmentation result is not desired to be too perfect, since information other than nuclei, such as cytoplasm and shape of the whole cell, also contributes to the heat map.

1 $$L_{{{\text{dice}}}} = 1 - 2 \times \frac{{\mathop \sum \nolimits_{i \in I} p_{i} y_{i} + \epsilon }}{{\mathop \sum \nolimits_{i \in I} p_{i} + \mathop \sum \nolimits_{i \in I} y_{i} + \epsilon }}$$

### Realization of MIL in the MobileNet V2

The main guiding methodology in our work is MIL, which is a kind of weak supervised learning method to deal with a lack of annotations. All the tiles could be fed to train the learning model. However, such an approach has serious drawbacks during classification. In many cases, the content of one small tile conflicts with the label of the original pathological image, especially in HCC cases with great heterogeneity. To solve this problem, MobileNet V2 was developed using MIL for training only on tile collections that carried a label for the associated whole-slide image. In this way, we could use MIL to take information on features from every tile. Instead of annotating each tile with its ancestor’s label and dumping it into the network directly, we packed all the tiles into a bag with a label identical to the original pathological image. Each bag, which represents one pathological image, was then passed through a trained neural network to calculate the scores of each tile in the bag, and an aggregational function was used to produce a weight-average score for the whole bag. By setting a threshold, the pathological image was classified into a certain class.

Each 224 × 224 tile was color-normalized using the method described by Vahadane et al. [28]. After nuclei segmentation, the color-normalized RGB tiles were then concatenated with their heat map in channel level to produce a four-channel tile. Then, these bags of four-channel tiles were dumped into a feature extractor, which is a MobileNet V2 model, and the score of each tile was calculated. A generalized mean was used as the aggregation function since it could keep the extremes while taking into account the average. The aggregation function reads as (2), where $p$ is a hyperparameter.

2 $$S = \left( {\sum s_{i}^{p} } \right)^{\frac{1}{p}}$$

The output of the aggregation function, which represents the score of the pathological image, was activated by a sigmoid function and compared with a given threshold $t$, where $t$ is also a hyperparameter. Based on this, the image was finally classified into a certain class.

### Training strategy

During the training process, we deployed a decay learning rate, which was initiated with 0.0001 and halves every 10 epochs. Due to the limitation of GPU memory, the training batch size could only be set to 1. Besides, threshold $t$ was 0.4457, and aggregation function $p$ was 3. Cross-entropy with L2 regulation (3)–(4) was selected as the loss function, and regularized factor $\alpha$ was 0.02.

3 $$p_{i} = \left\{ {\begin{array}{*{20}c} {\left( {\frac{{t - s_{i} }}{t} + 1} \right)*0.5,s_{i} \le t } \\ {\left( {1 - \frac{{s_{i} - t}}{1 - t}} \right)*0.5, s_{i} > t} \\ \end{array} } \right.$$

4 $$L\left( {\left\{ {p_{i} } \right\}, \left\{ {y_{i} } \right\}, W} \right) = - \frac{1}{N}\sum \left[ {y_{i} *\log \left( {p_{i} } \right) + \left( {1 - y_{i} } \right)*\log \left( {1 - p_{i} } \right)} \right] + \alpha \mathop \sum \limits_{{w_{i} \in W}} w_{i}^{2}$$

### Analysis on tiles with high predictive value

To deepen our understanding of features related to tumor aggressiveness, tiles having high and low-risk scores were retrieved for further in-depth analysis. Altogether four histological features of tumoral liver tissues have been systematically documented.

### Statistical analysis

Sample size for survival analysis was determined by power analysis using PASS15.0.5 software. For the validation dataset, the type I error is controlled for RFS at *α* = 0.01 (two-sided) with the power of 90% (i.e., Hazard Ratio (HR) is set at 2.0), sample size is 137 (low risk: 68 vs high risk: 69). The sample size of two validation dataset (LT and TCGA) provides more than 90% power to detect a difference for RFS. Log-rank tests were performed to compare the stratification of patients into subgroups in terms of survival distribution. Time-dependent accuracy (at the best Youden index) and AUC (area under curve) methods were adopted as an index to assess the proposed model’s performance and the baseline clinical, biological, and pathological features [29]. Net reclassification improvement (NRI) has been widely used to assess the performance of a prediction model by comparing the new variables with the established model [30]. NRI was used to query the additional effect of our model on survival prediction. Statistical analyses were carried out with R (version 3.6.0) using ggplot2, survival, and Survminer packages. The training and deployment of CNNs were conducted with Python using a standard desktop workstation (Nvidia Tesla P40 GPUs each with 24GM memory). *p* value < 0.05 indicated statistical significance.

## Results

### Patient characteristics and model development

The nucleus map set was used to train U-net to capture localized nuclear architectural information (*n* = 120). The other three sets that were used for training and validation were: (1) 552 patients from the train set that joined in the development of the model, (2) 144 patients from the LT set, and (3) the TCGA set (*n* = 302) that was used to externally validate the model. Patients from the train set exhibited obvious outcomes (good: 274, bad: 278) and were enrolled for obtaining definite facts. The patients’ demographics are presented in Table 1.

**Table 1:** Baseline characteristics in the nucleus map set, train set, LT set, and TCGA set

Variables	Nucleus map set (*n* = 120)	Train set (*n* = 552)	LT set (*n* = 144)	TCGA set (*n* = 302)
Age (year)	59 (49–65)	55 (47–63)	52 (45–58)	60 (51–68)
Gender (male)	104 (86.7%)	478 (86.6%)	130 (90.3%)	208 (68.9%)
AFP (ng/ml)	34.7 (6.6- 708.0)	76.5 (7.2–888.0)	49.3 (7.7–1418.3)^a^	11.0 (4.0–231.5)^b^
Grade				
G1	15 (12.5%)	32 (5.8%)	3 (2.1%)	43 (14.2%)
G2	54 (45.0%)	243 (44.0%)	42 (29.2%)	142 (47.0%)
G3	42 (35.0%)	217 (39.3%)	35 (24.3%)	103 (34.1%)
G4	9 (7.5%)	54 (9.8%)	0 (0.0%)	10 (3.3%)
Missing	0 (0.0%)	6 (1.1%)	64 (44.4%)	4 (1.3%)
Total tumor size				
< 5 cm	79 (65.8%)	334 (60.5%)	35 (24.3%)	
≥ 5 cm	41 (34.2%)	210 (38.0%)	109 (75.7%)	
Missing	0 (0.0%)	8 (1.4%)	0 (0.0%)	302 (100.0%)
Tumor number				
Single	108 (90.0%)	479 (86.8%)	52 (36.1%)	
Multiple	12 (10.0%)	67 (12.1%)	92 (63.9%)	
Missing	0 (0.0%)	6 (1.1%)	0 (0.0%)	302 (100.0%)
Stage_AJCC				
Stage I	90 (75.0%)	335 (60.7%)	25 (17.4%)	144 (47.7%)
Stage II	25 (20.8%)	145 (26.3%)	34 (23.6%)	66 (21.9%)
Stage III	5 (4.2%)	53 (9.6%)	63 (43.8%)	69 (22.8%)
Stage IV	0 (0.0%)	12 (2.3%)	21 (14.6%)	3 (1.0%)
Missing	0 (0.0%)	7 (1.3%)	1 (0.7%)	20 (6.6%)

Data are median (IQR) or *n* (%)

^a^Not available = 1

^b^Not available = 71

**Fig. 1:**
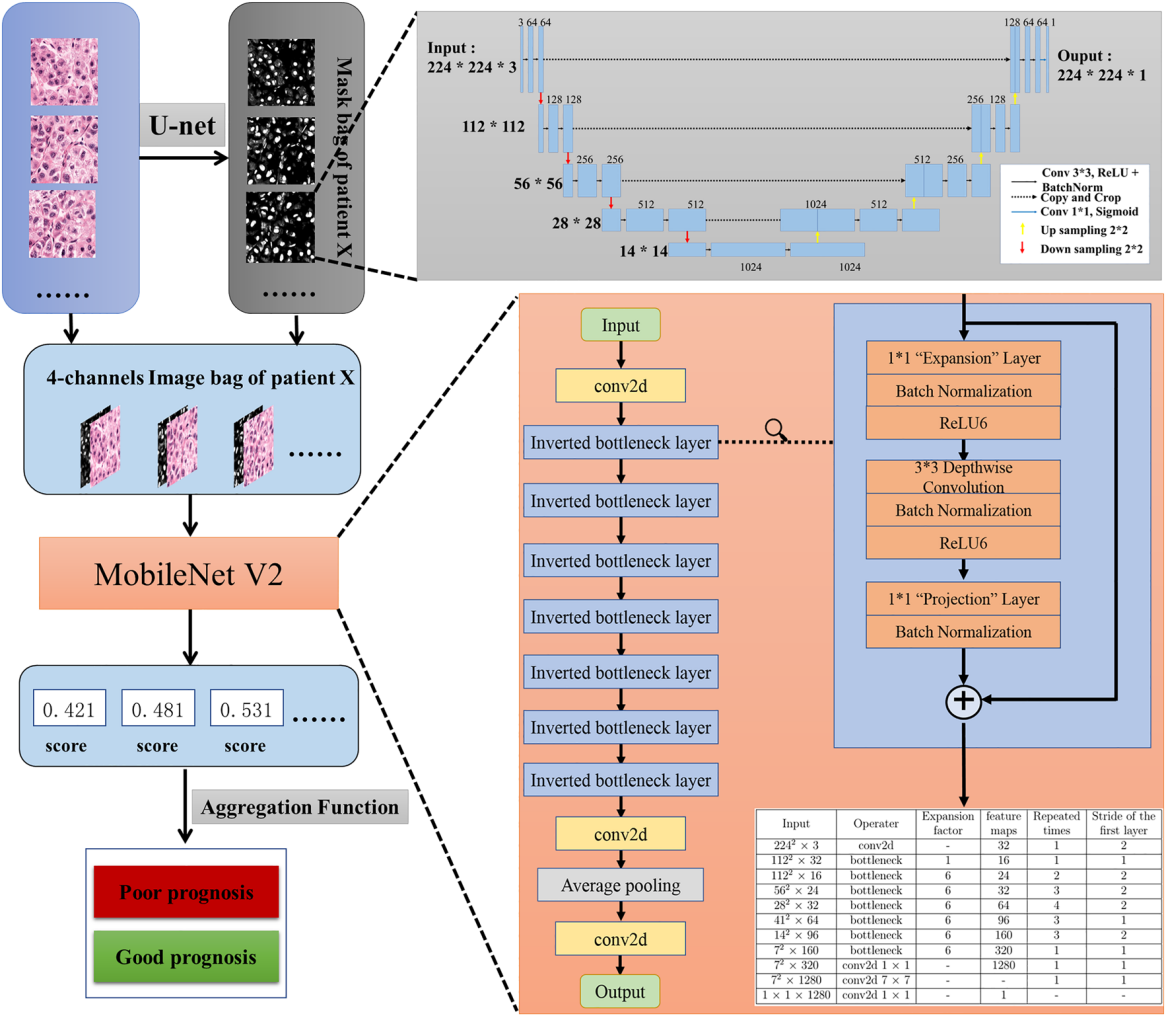
The pipeline for MobileNetV2_HCC_Class. From the small image patches in 224 × 224 pixels of the train set, the heat map of nuclei segmentation for each tile was obtained using a pre-trained U-net. The heatmap of nuclei segmentation and the color-normalized RGB tiles were concatenated at channel level, and a four-channel tile was obtained. Bags containing four-channel tiles were then dumped into a feature extractor of the MobileNetV2 model. A generalized mean with a sign was used as the aggregation function

First, we used an image segmentation model to get the heat map of nuclei segmentation for each tile. This segmentation model was a U-net neural network trained using the nucleus map set. The loss function was Dice and the final Dice Score for the nucleus map set could reach 82%. The segmentation result was not desired to be too precise, since information other than nuclei, such as cytoplasm and shape of the whole cell, was also accountable in the heatmap. A total of 57,415 tiles (small image patches with 224 × 224 pixels) were extracted from the train set (good: 28,534, poor: 28,881). The pre-trained U-net was used to get a heat map of nuclei segmentation for each tile before finally training our model. We concatenated the heat map of nuclei segmentation and the color-normalized RGB tiles at channel level and produced a four-channel tile. The bags containing four-channel tiles were then dumped onto a feature extractor of the MobileNet V2 model. We used a generalized mean with a sign as the aggregation function since it could keep the extremes while simultaneously taking the average into account. The output of the aggregation function, which represents the score of the pathological image was activated using a sigmoid function and then compared with a given threshold of 0.4457, where 0.4457 is a hyperparameter. Finally, the images are classified into certain a class based on their scores. The pipeline for MobileNet V2 HCC classification (MobileNet V2_HCC_Class) is shown in Fig. 1.

### The model generalized to LT for the HCC dataset

**Fig. 2:**
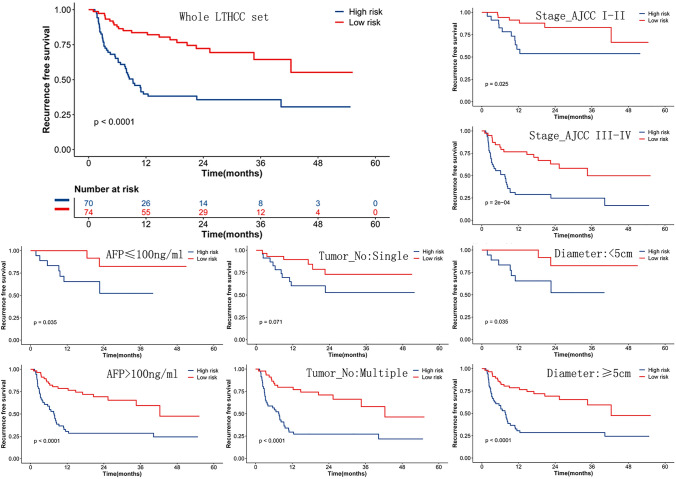
Prognostic value of MobileNetV2_HCC_Class in LT set and the stratification of common prognostic variables. MobileNetV2_HCC_Class categorized patients into low-risk and high-risk subgroups. The prognostic value for MobileNetV2_HCC_Class was conservative, even following the stratification of common clinical and pathological variables. AFP: alpha-fetoprotein, Tumor No: tumor number, Diameter: total tumor diameter

The output of our neural networks could categorize patients into low-risk and high-risk subgroups. In the LT set, 144 patients with complete follow-up data were included, of which 65 patients relapsed during follow-up. The available variables for analysis are age at diagnosis, gender, serum alpha-fetoprotein (AFP), Child–Pugh score, the model for end-stage liver disease (MELD), tumor size, tumor number, grade, and tumor stage according to the American Joint Committee on Cancer (Stage AJCC). Univariable analyses indicated that the variables AFP, tumor size, grade, tumor number, and Stage AJCC were associated with a shorter RFS (Table S1). Tiles from the tissue array of these patients were retrieved and processed under the proposed model. The MobileNetV2_HCC_class was a strong predictor of RFS in the whole LT set and was even capable of stratification for other common prognostic features (Stage AJCC, AFP, tumor number, and tumor size) (Fig. 2).

**Fig. 3:**
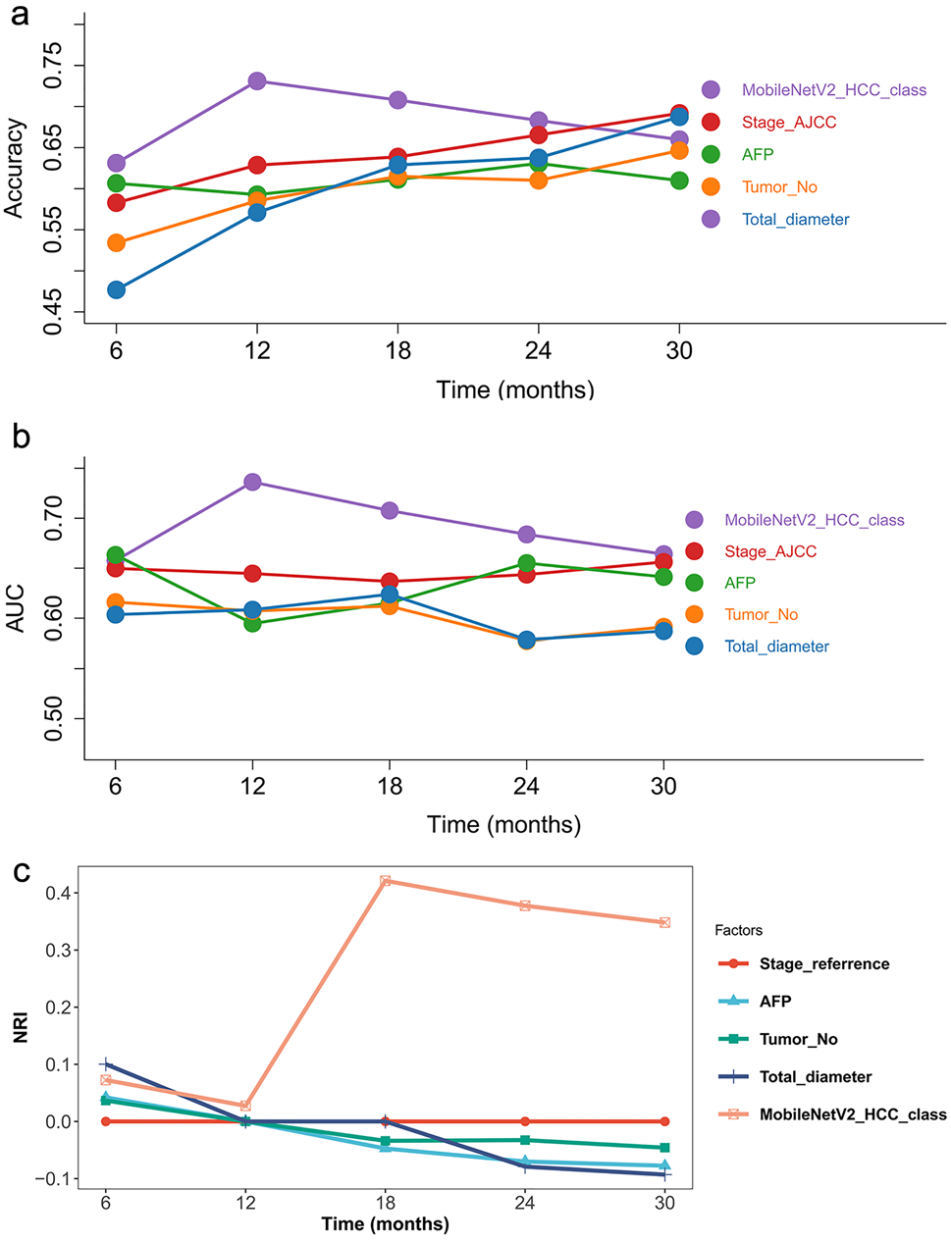
The performance of different risk factors for tumor recurrence after LT. The time-dependent accuracy (**a**) and AUC value (**b**) for different criteria based on tumor recurrence. NRI (**c**) according to different factors compared with the Stage_AJCC. Stage AJCC: the American Joint Committee on Cancer, AFP: serum alpha-fetoprotein, Tumor_No: tumor number, Total_diameter: total diameter of the tumor

Multivariate analyses showed that the MobileNetV2_HCC_class had an independent prognostic value (HR = 3.44 (2.01–5.87), *p* < 0.001) after adjusting known prognostic markers remarkable in the univariable analyses, such as Stage AJCC, AFP, tumor number, and tumor size (Fig. S1). The time-dependent accuracy and AUC curves are depicted in Fig. 3a and b. During the entire course of the 3-year follow-up, the MobileNetV2_HCC_class maintained relatively higher accuracy and AUC values than the other factors in the first 2 years after LT (Tables 2 and 3). Next, we assessed the contribution of MobileNetV2_HCC_class to the established predictors for RFS in the independent LT set. Stage_AJCC was well established based on the TNM staging system and could identify patient groups with substantially different RFS in the LT set (Fig. S2). Therefore, we examined if the addition of the MobileNetV2_HCC_class as well as the other independent factors (AFP, tumor size, tumor number) to Stage_AJCC could improve their abilities for risk stratification. It was found that MobileNetV2_HCC_class exhibited better net benefits for the Stage_AJCC beyond other previously established factors (Fig. 3c). The category-free NRI of the supplementing Stage_AJCC with MobileNetV2_HCC_class for predicting 2-year RFS was 37.8% (95% CI 20.8–55.5). The time-dependent NRI of HCC patients according to different factors in comparison with the Stage_AJCC are shown in Table 4.

**Table 2:** The time-dependent accuracy values (95% CI) in LT set

Time point	MobileNetV2_ HCC_class	Stage_AJCC	AFP	Tumor_No	Total_diameter
6 months	0.631 (0.620–0.642)	0.583 (0.574–0.592)	0.607 (0.598–0.616	0.534 (0.524–0.544)	0.477 (0.469–0.485)
12 months	0.731 (0.722–0.741)	0.629 (0.620–0.637)	0.593 (0.585–0.601)	0.585 (0.576–0.594)	0.571 (0.563–0.578)
18 months	0.708 (0.698–0.718)	0.639 (0.630–0.647)	0.611 (0.603–0.619)	0.615 (0.606–0.624)	0.629 (0.622–0.636)
24 months	0.683 (0.672–0.694)	0.665 (0.656–0.675)	0.631 (0.621–0.640)	0.610 (0.600–0.620)	0.638 (0.629–0.647)
30 months	0.660 (0.647–0.672)	0.692 (0.681–0.702)	0.610 (0.599–0.621)	0.646 (0.636–0.657)	0.687 (0.678–0.697)

*Stage AJCC* the American Joint Committee on Cancer, *AFP* serum alpha-fetoprotein, *Tumor_No* tumor number, *Total_diameter* total diameter of the tumor

**Table 3:** The time-dependent AUC values (95% CI) in LT set

Time point	MobileNetV2_ HCC_class	Stage_AJCC	AFP	Tumor_No	Total_diameter
6 months	0.658 (0.645–0.670)	0.650 (0.641–0.659)	0.663 (0.649–0.677)	0.616 (0.606–0.626)	0.604 (0.595–0.612)
12 months	0.736 (0.726–0.746)	0.645 (0.636–0.653)	0.595 (0.585–0.605)	0.607 (0.598–0.616)	0.609 (0.601–0.616)
18 months	0.708 (0.698–0.717)	0.637 (0.628–0.645)	0.615 (0.606–0.625)	0.612 (0.603–0.621)	0.624 (0.617–0.631)
24 months	0.684 (0.674–0.694)	0.644 (0.634–0.653)	0.655 (0.645–0.665)	0.578 (0.568–0.588)	0.579 (0.570–0.588)
30 months	0.664 (0.653–0.675)	0.656 (0.647–0.666)	0.641 (0.630–0.652)	0.591 (0.581–0.601)	0.587 (0.578–0.596)

*Stage AJCC* the American Joint Committee on Cancer, *AFP* serum alpha-fetoprotein, *Tumor_No* tumor number, *Total_diameter* total diameter of the tumor

**Table 4:** The time-dependent NRI of patients according to different factors compared with the Stage_AJCC (NRI with 95% CI) in LT set

Time point	Stage_AJCC	AFP	Tumor_No	Total_diameter	MobileNetV2_ HCC_class
6 months	Reference	0.042 (− 0.119–0.192)	0.037 (− 0.120–0.180)	0.100 (0.048–0.157)	0.072 (− 0.106–0.233)
12 months	Reference	0.000 (0.000–0.000)	0.000 (0.000–0.000)	0.000 (0.000–0.000)	0.027 (− 0.105–0.165)
18 months	Reference	− 0.047 (− 0.159–0.066)	− 0.034 (− 0.186–0.119)	0.000 (0.000–0.000)	0.421 (0.274–0.577)
24 months	Reference	− 0.070 (− 0.169–0.037)	− 0.033 (− 0.192–0.134)	− 0.079 (− 0.227–0.078)	0.378 (0.208–0.555)
30 months	Reference	− 0.077 (− 0.193–0.029)	− 0.046 (− 0.197–0.130)	− 0.093 (− 0.248–0.086)	0.348 (0.180–0.510)

*Stage AJCC* the American Joint Committee on Cancer, *AFP* serum alpha-fetoprotein, *Tumor_No* tumor number, *Total_diameter* total diameter of the tumor

### The model generalized to the TCGA dataset

**Fig. 4:**
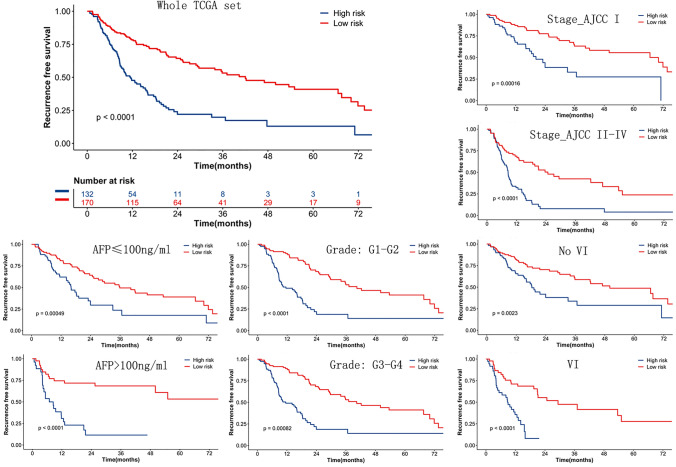
Prognosis of MobileNetV2_HCC_Class in TCGA set and the stratification of common baseline variables. MobileNetV2_HCC_Class predicts RFS while also following the stratification of other common baseline variables. Stage AJCC: the American Joint Committee on Cancer, AFP: serum alpha-fetoprotein, VI: vascular invasion

The robustness of our model was evaluated on an independent series from the TCGA. 302 patients satisfied the inclusion criteria, and 165 of them with recurrence were recorded. The slides were gathered from various centers. The available variables that were entered for analysis are age at diagnosis, age, gender, AFP, vascular invasion, stroma tumor ratio (STR), tumor-infiltrating lymphocyte (TIL), grade, and Stage AJCC. The clinical, biological, and pathological feature most related to a shorter survival should be the AJCC stage in univariable analyses (Table S5). Tiles from WSIs of the 302 patients were retrieved and processed under the proposed model. In the TCGA set, MobileNetV2_HCC_class predicted the RFS while also following the stratification of other significant prognostic features like Stage AJCC, AFP, grade, or vascular invasion (Fig. 4).

**Fig. 5:**
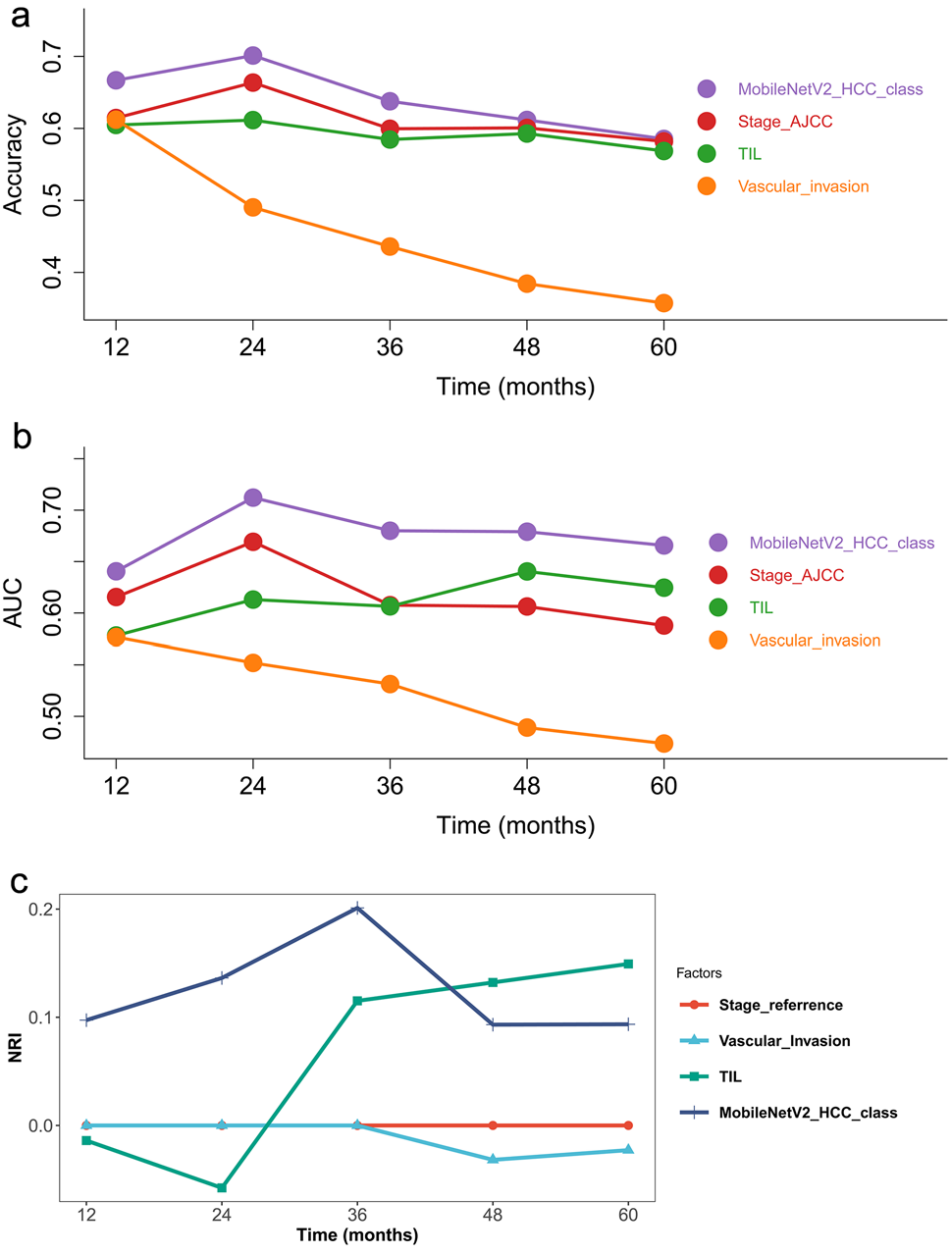
Performance of different risk factors in tumor recurrence after resection. The time-dependent accuracy (**a**) and AUC value (**b**) for different criteria based on tumor recurrence. NRI (**c**) according to different factors compared with the Stage_AJCC. Stage AJCC: the American Joint Committee on Cancer, TIL: tumor-infiltrating lymphocyte

The classifier seemed strong during multivariable analysis (HR = 2.55 (1.64–3.99), *p* < 0.001), upon adjusting the known prognostic markers remarkable in univariable analyses, such as Stage AJCC, AFP, grade, and vascular invasion (Fig. S3). The results show that the model can capture complicated non-redundant patterns in which baseline variables influence HCC patients’ survival. The time-dependent accuracy and AUC curves are depicted in Fig. 5a and b. During the entire course of the 6-year follow-up, the MobileNetV2_HCC_class maintained relatively higher accuracy and AUC values than other factors after HCC resection (Tables S3 and S4). Stage_AJCC was the prognostic indicator of RFS in the TCGA set (Fig. S4). NRI analysis was also performed in TCGA set. Similarly, MobileNetV2_HCC_class exhibited better net benefits for the Stage_AJCC beyond other independent factors (Fig. 5c). The improvement of MobileNetV2_HCC_class was obvious and the category-free NRI of the supplementing Stage_AJCC with MobileNetV2_HCC_class for predicting 3-year RFS was 20.1% (95% CI 5.7–47.1). The time-dependent NRI of HCC patients according to different factors in comparison with the Stage_AJCC are shown in Table S5.

### Histological analysis of tiles

**Fig. 6:**
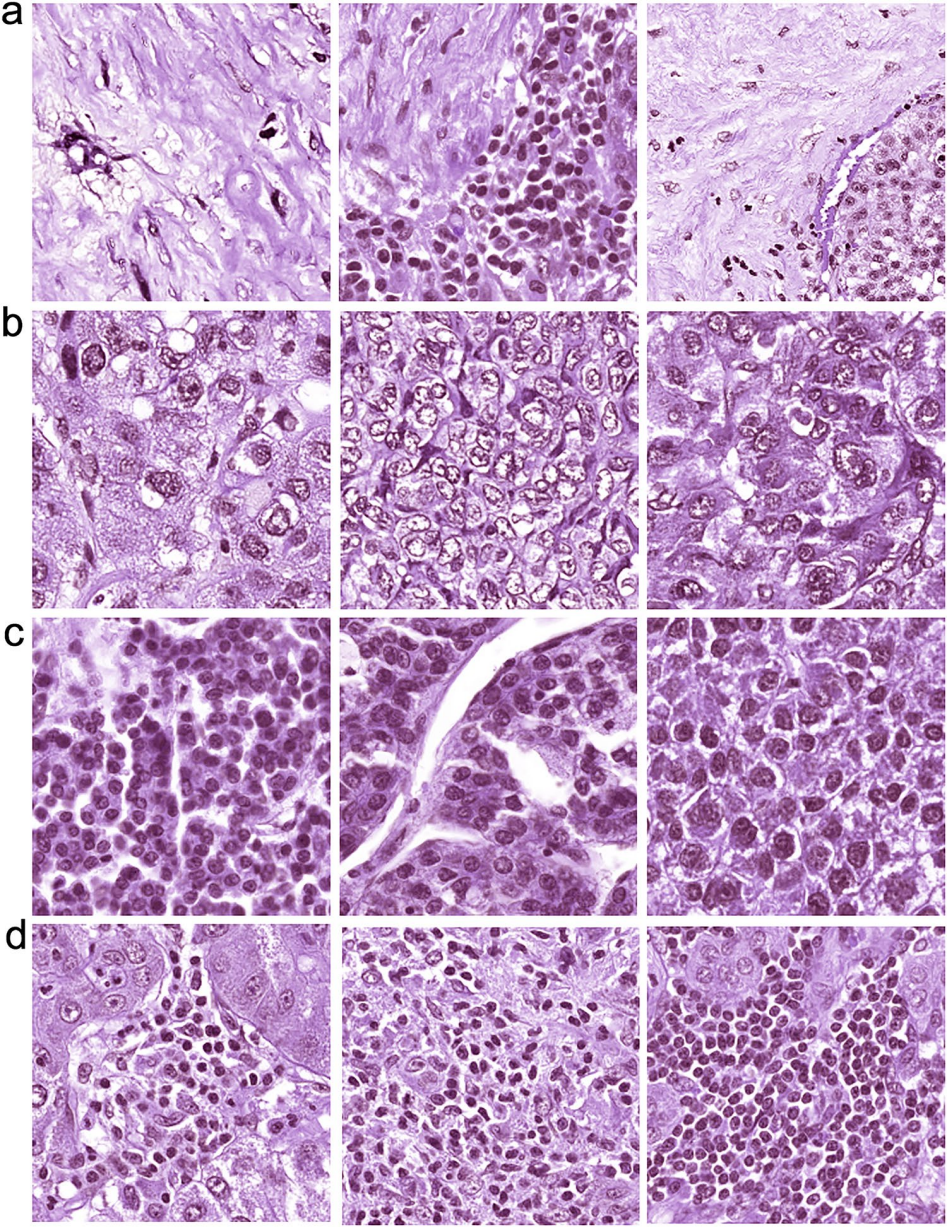
Typical tiles were found to have low or high risks usingMobileNetV2_HCC_Class. Four hundred tiles with the highest predictability were investigated. The features used to predict high recurrence risk were stroma (**a**), cellular atypia (**b**), and nuclear hyperchromasia (**c**). The feature indicating low recurrence risk was the existence of immune cells (**d**)

The MobileNetV2_HCC_class could extract tiles with the highest predictability from thousands of tiles. The prime histological features related to recurrence could be surveyed by retrieving 400 tiles with the highest predictability (high recurrence risk: 200, low recurrence risk: 200) among 302 patients of the TCGA with MobileNetV2_HCC_class. Four such histological features were found from tumoral areas. The presence of stroma, high degree of cytological atypia, and nuclear hyperchromasia were related to high risk (*p* = 0.0003, *p* = 0.0010, *p* = 0.0012, respectively), while immune cell infiltration was associated with low risk (*p* = 0.0019) (Fig. 6, Table S6). The above findings show that the proposed deep learning model detects established histological patterns related to recurrence among HCC patients.

## Discussion

Based on the latest developments in deep learning, this study proposed the use of MobileNetV2_HCC_class for automatic prognosis prediction in HCC patients. This would enable automated analyses of standard histological sections stained with H&E. These algorithms exhibit a higher accuracy in prognosis prediction relative to classical clinical, biological, and pathological features.

Deep learning-driven methods in medical image processing have proven to be extremely useful in standardizing cancer diagnosis as well as in improving patient stratification [19, 31]. Recently, a pioneering survey reported that deep learning-based models can detect and categorize lung cancer cases with accuracy similar to that of pathologists [20]. Previous studies suggest that deep learning is highly efficient in developing markers, which utilize basic morphology for the prediction of outcomes in cancer patients [32, 33]. A deep learning-based model developed by Coudray et al. could even predict six of the most frequent genetic alterations directly from the slides [20]. In cases of gastrointestinal cancer, a deep learning-based model could directly estimate microsatellite instability based on just histological images [27]. Kather et al. also reported that a CNN could extract the tumor components and predict patient survival directly from histology images [21]. Saillard et al. predicted the survival of HCC patients by extracting features from images using a pre-trained CNN, following which the network selected 25 tiles having maximum and minimum scores to predict survival [22]. In our study, a different method was used to develop the MobileNetV2_HCC_class to improve the prediction of prognosis in HCC patients treated by surgical resection and LT. The innovative features of our method were: (1) random tiles were used for each patient, like Skrede et al. [17], (2) the MobileNet V2 was trained using MIL, which allowed for training on tile collections labeled with the associated whole-slide image, and (3) the use of nuclear architectural information in building of the model, which proved to be efficient for cancer grading and prediction of patient outcomes [24]. Genetic instability was demonstrated through diversifying nuclear shape and texture, which had a major effect on metastasis and proliferation that might lead to cancer recurrence. The MobileNetV2_HCC_class proved to be a strong predictor of RFS in HCC patients treated with resection or LT and generalized in the TCGA set across different centers.

It is well established that molecular and/or genetic features can predict the survival of patients with HCC [34, 35]. Chaudhary et al. had adopted deep-learning methods for RNA sequencing and methylation data from the TCGA database and successfully predicted HCC survival in multiple patient cohorts. High-throughput gene expression profiling/sequencing techniques are restrictive due to high cost and poor reproducibility clinically. The proposed method uses merely the histological slides that are routinely available at surgical treatment centers. We provide additional evidence suggesting that CNNs learning from pathology slides will improve precision medicine. Indeed, our models outperformed all other common clinical or pathological features for predicting survival. Pathology images from centers around the world can be accumulated, which would further improve the performance of the current deep learning model. With the well-developed prediction models, they would likely become more widely applied to support clinical decision-making, and can benefit patients by stratifying risks and guiding treatment options, as well as by avoiding ineffective or unnecessary treatments. Additionally, the processing and computing time in this approach is brief enough to avoid delay in therapy. Therefore, this method facilitates the easy application of the risk stratification system clinically. However, this study is limited by the lack of interpretability. CNNs are generally seen as “black boxes” [36]. This is particularly true for image analysis, and the limitation of this phenomenon is an active area of research. Data are processed through complex layers of CNNs, and it is difficult to identify the most relevant features used by trained models for final classification. We extracted the most pertinent tiles and did the subsequent analysis. We show that the classification obtained is at least partly based on known pathological features associated with the prognosis of tumor, such as the presence of stroma, a high degree of cytological atypia, nuclear hyperchromasia, and a lack of immune cell infiltration [37]. However, these features are just what pathologists know strongly linked to a high risk of poor survival. We thus believe that some other important features which cannot be recognized or microstructural features that cannot be consistently identified by the naked eye, but these features which could potentially be reflective of tile classification may be ignored. The so-called high risk or low risk are the overall output result of tiles via considering all risk features on tiles learned by CNNs. That maybe can explain why low-risk tiles contain some high-risk features, although the ratio is small. The proposed deep learning model detects not all but the majority of established histological patterns related to recurrence in HCC patients.

To sum up, we successfully built a prognostic model for clinical use based on deep learning approaches applied on histological slides from patients. The model was widely assessed among independent patient populations receiving different types of treatments and gave consistently excellent results across the classical clinical, biological, and pathological features. The proposed CNN-based approach can potentially improve patient prognosis evaluation and help guide clinicians in their decision-making process about the use of adjuvant therapy on their patients.

## Supplementary Information

Below is the link to the electronic supplementary material.

Supplementary file1 (DOCX 1975 KB)

## Data Availability

The data that support the findings of this study are available from the corresponding author on reasonable request.

## Declarations

### Code availability

The U-net or MobileNet V2 neural networks are available from the corresponding author on request.
