# Supplementary material for: Deep learning for prediction of hepatocellular carcinoma recurrence after resection or liver transplantation: a discovery and validation study
Source: Hepatol Int. 2022 Mar 29;16(3):577–89. doi: 10.1007/s12072-022-10321-y (PMC9174321; doi:10.1007/s12072-022-10321-y)
Supplement: Supplementary file 1 — Supplementary file1 (DOCX 1975 KB) [file 12072_2022_10321_MOESM1_ESM.docx]

**Supplemental Material**

**Supplemental Figures**


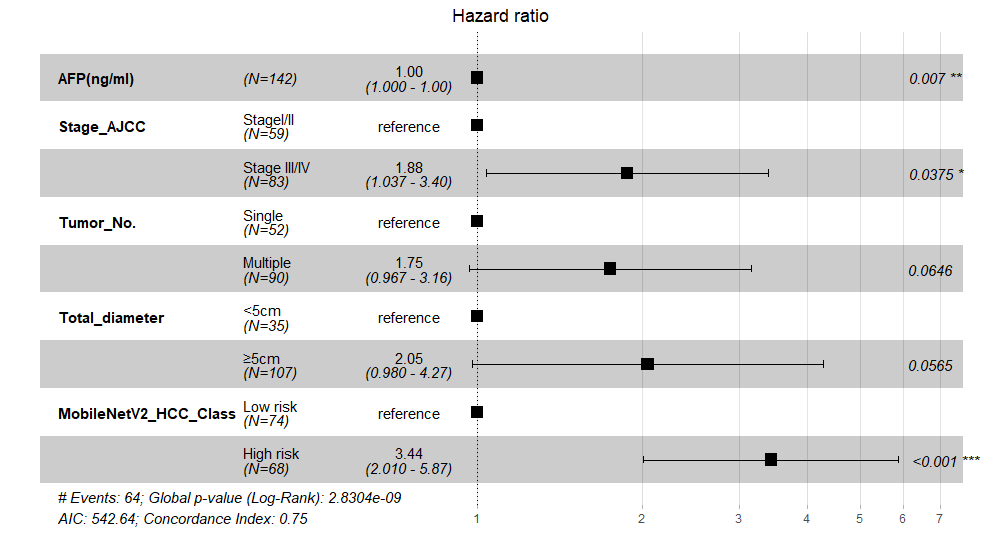


Figure S1. Multivariate analyses of risk factors for tumor recurrence after LT in LT set. Stage AJCC, the American Joint Committee on Cancer; AFP, serum alpha fetoprotein; Tumor_No, tumor number; Total_diameter, total diameter of the tumor.


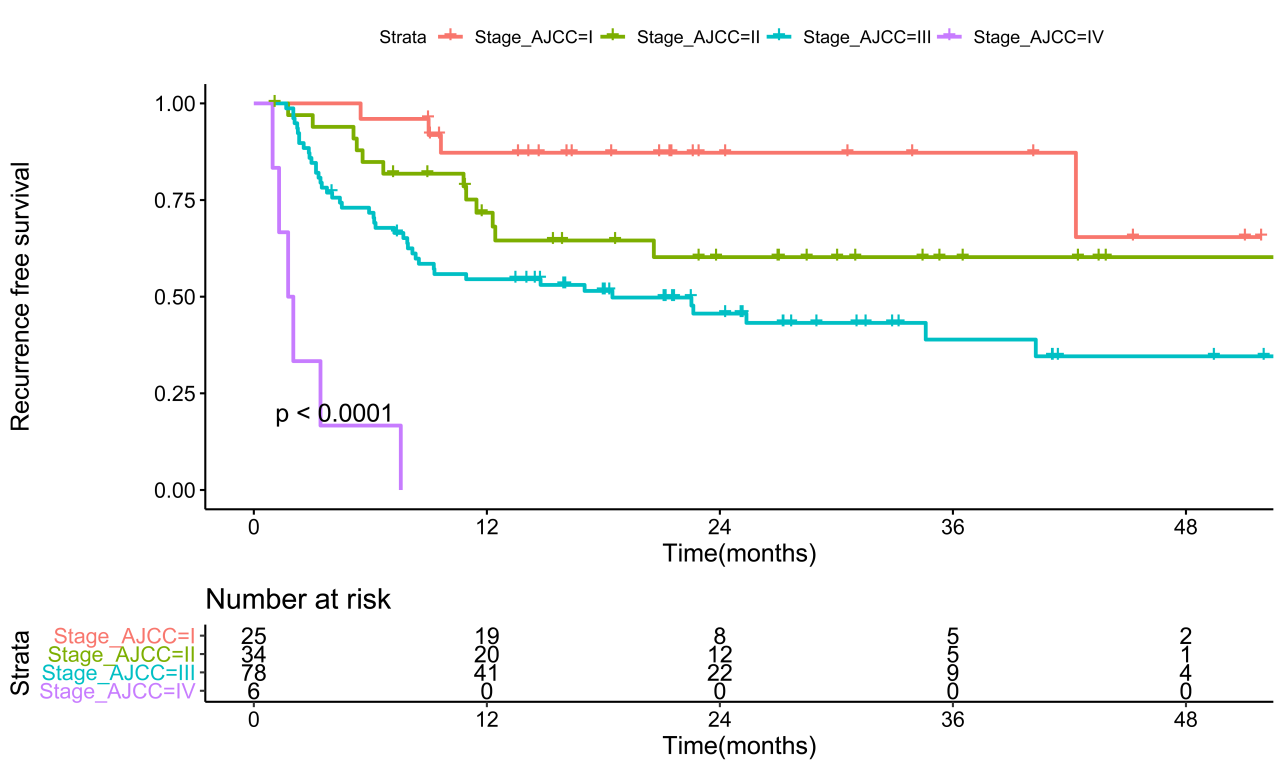


Figure S2. The recurrence free survival analysis according to the Stage_AJCC in the LT set.


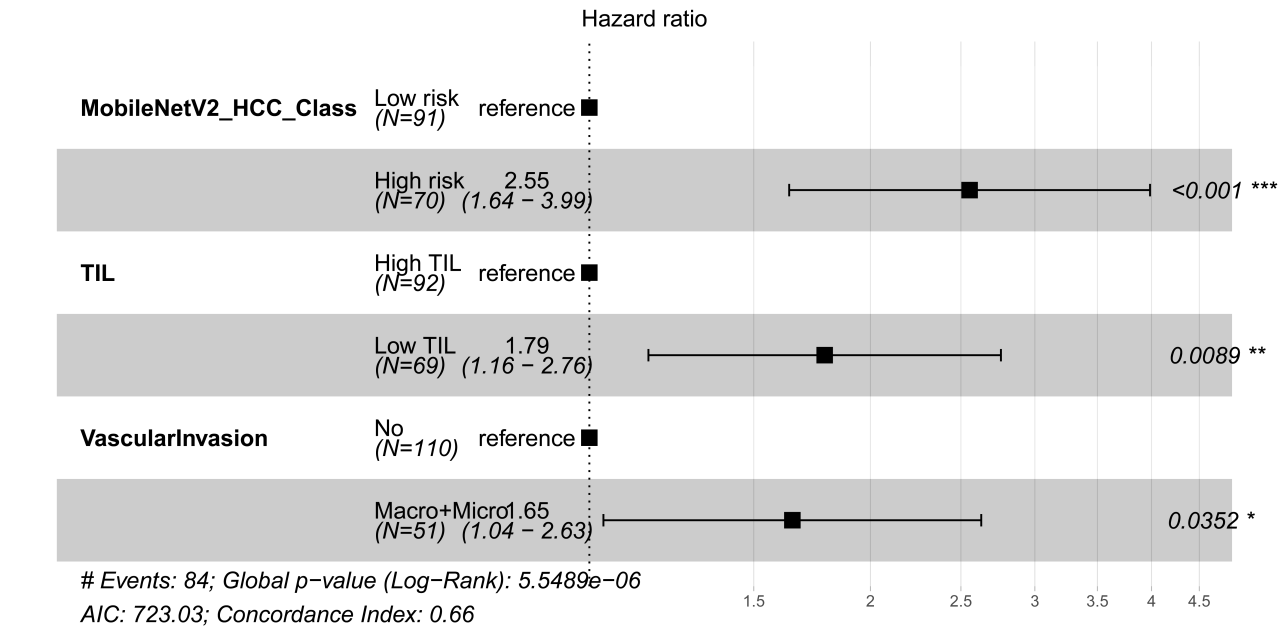


Figure S3. Multivariate analyses of risk factors for tumor recurrence after operation in TCGA set. AFP, serum alpha fetoprotein; TIL, tumor infiltrating lymphocyte; Vascular_invasion, macrovascular and microvascular invasion.


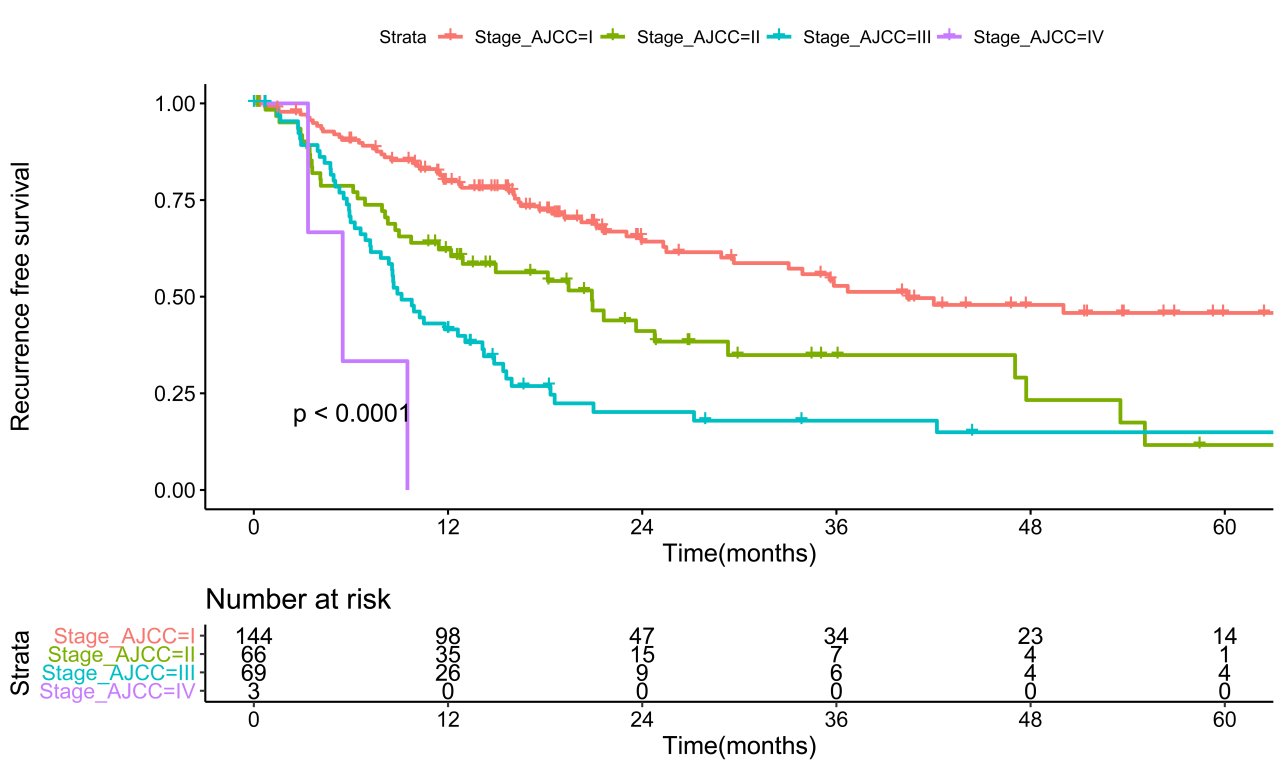


Figure S4. The recurrence free survival analysis according to the Stage_AJCC in the TCGA set.

**Supplemental Tables**

Table S1. Univariable recurrence free survival analyses in LT set (n = 144).

| Variables | HR | z | P value |
| --- | --- | --- | --- |
| Age (years) | 0.988(0.963-1.014) | -0.924 | 0.356 |
| Gender (female vs male) | 0.564(0.205-1.553) | -1.108 | 0.268 |
| CHLID | 1.021(0.910-1.144) | 0.352 | 0.725 |
| MELD | 0.994(0.974-1.015) | -0.569 | 0.569 |
| AFP (positive vs negative) | 1.338(0.798-2.244) | 1.104 | 0.270 |
| MobileNetV2_HCC_Class (high risk vs low risk) | 3.130 (1.857-5.277) | 4.283 | 1.846E-05 |
| Tumor_No (single vs multiple) | 0.459(0.261-0.808) | -2.697 | 0.007 |
| Total diameter (≥5cm vs <5cm) | 2.502(1.235-5.066) | 2.547 | 0.011 |
| Stage_AJCC (Stage I/II vs Stage III/IV) | 0.355(0.202-0.626) | -3.582 | <0.001 |
| Grade (well/moderate vs poor) | 0.517(0.269-0.992) | -1.983 | 0.047 |

Abbreviations: CHILD, Child-Pugh score; MELD, Model for end-stage liver disease; AFP, serum alpha fetoprotein; Tumor_No, tumor number; Stage AJCC, the American Joint Committee on Cancer.

Table S2. Univariable recurrence free survival analyses in TCGA set (n = 302).

| Variables | HR | z | P value |
| --- | --- | --- | --- |
| Age (years) | 0.996(0.984-1.008) | -0.696 | 0.486 |
| Gender (female vs male) | 0.859(0.621-1.188) | -0.918 | 0.359 |
| AFP (positive vs negative) | 1.380(0.966-1.971) | 1.771 | 0.077 |
| Vascular invasion (no vs yes) | 0.544(0.379-0.779) | -3.325 | 0.001 |
| TIL (low vs high) | 1.577(1.101-2.259) | 2.485 | 0.013 |
| STR (low vs high) | 0.692(0.508-0.943) | -2.335 | 0.020 |
| Stage AJCC (II vs I) | 1.937(1.287-2.915) | 3.172 | 0.002 |
| Stage AJCC (III/IV vs I) | 3.079(1.120-4.471) | 5.910 | 3.423E-09 |
| MobileNetV2_HCC_Class (high risk vs low risk) | 2.724(1.982-3.746) | 6.170 | 6.833E-10 |

Abbreviations: AFP, serum alpha fetoprotein; STR, stroma tumor ratio; TIL, tumor infiltrating lymphocyte; Stage AJCC, the American Joint Committee on Cancer; Vascular_invasion, macrovascular and microvascular invasion.

Table S3. The time-dependent accuracy values (95%CI) in TCGA set.

| Time point | MobileNetV2_HCC_class | Stage_AJCC | TIL | Vascular_invasion |
| --- | --- | --- | --- | --- |
| 12 months | 0.667(0.657-0.676) | 0.614(0.605-0.624) | 0.605(0.595-0.614) | 0.612(0.604-0.620) |
| 24 months | 0.701(0.691-0.712) | 0.664(0.653-0.674) | 0.612(0.600-0.624) | 0.490(0.480-0.500) |
| 36 months | 0.638(0.626-0.649) | 0.600(0.587-0.612) | 0.585(0.572-0.598) | 0.436(0.425-0.447) |
| 48 months | 0.612(0.600-0.624) | 0.601(0.587-0.615) | 0.593(0.580-0.606) | 0.384(0.373-0.396) |
| 60 months | 0.585(0.573-0.598) | 0.582(0.568-0.596) | 0.569(0.556-0.582) | 0.357(0.346-0.368) |

Abbreviations: Stage AJCC, the American Joint Committee on Cancer; TIL, tumor infiltrating lymphocyte; Vascular_invasion, macrovascular and microvascular invasion.

Table S4. The time-dependent AUC values (95%CI) in TCGA set.

| Time point | MobileNetV2_HCC_class | Stage_AJCC | TIL | Vascular_invasion |
| --- | --- | --- | --- | --- |
| 12 months | 0.641(0.631-0.650) | 0.616(0.605-0.626) | 0.578(0.568-0.588) | 0.577(0.568-0.585) |
| 24 months | 0.712(0.702-0.722) | 0.669(0.658-0.680) | 0.613(0.602-0.625) | 0.552(0.543-0.561) |
| 36 months | 0.680(0.668-0.692) | 0.608(0.595-0.620) | 0.606(0.593-0.620) | 0.531(0.521-0.542) |
| 48 months | 0.679(0.665-0.693) | 0.606(0.591-0.622) | 0.640(0.626-0.655) | 0.489(0.476-0.502) |
| 60 months | 0.666(0.651-0.681) | 0.588(0.572-0.604) | 0.625(0.609-0.640) | 0.474(0.460-0.488) |

Abbreviations: Stage AJCC, the American Joint Committee on Cancer; TIL, tumor infiltrating lymphocyte; Vascular_invasion, macrovascular and microvascular invasion.

Table S5. The time-dependent NRI of patients according to different factors compared with the Stage_AJCC (NRI with 95% CI) in TCGA set.

| Time points | Stage_AJCC | Vasculaer invasion | TIL | MobileNetV2_HCC_class |
| --- | --- | --- | --- | --- |
| 12 months | Referrence | 0.000(0.000-0.000) | -0.014(-0.187-0.169) | 0.097(-0.100-0.295) |
| 24 months | Referrence | 0.000(0.000-0.000) | -0.058(-0.177-0.055) | 0.136(-0.065-0.307) |
| 36 months | Referrence | 0.000(0.000-0.000) | 0.115(-0.048-0.267) | 0.201(-0.057-0.471) |
| 48 months | Referrence | -0.032(-0.181-0.156) | 0.132(-0.002-0.282) | 0.093(-0.226-0.389) |
| 60 months | Referrence | -0.023(-0.174-0.164) | 0.149(0.007-0.343) | 0.094(-0.220-0.399) |

Abbreviations: Stage AJCC, the American Joint Committee on Cancer; TIL, tumor infiltrating lymphocyte; Vascular_invasion, macrovascular and microvascular invasion.

Table S6. Histological features in tiles associated with recurrence.

| Features | High risk | Low risk | P value |
| --- | --- | --- | --- |
| Presence of stroma | 25/200 | 4/200 | 0.0003 |
| High degree of cytological atypia | 36/200 | 11/200 | 0.0010 |
| Nuclear hyperchomasia | 28/200 | 7/200 | 0.0012 |
| Immune cell infiltration | 3/200 | 19/200 | 0.0019 |

**Supplemental Methods**

Original image preprocessing , tile cropping and normalization

The original image is not directly suitable as input to a convolutional neural network (CNN) because of limited GPU memory in commonly available hardware. We therefore made multiple non-overlapping regions of a fixed size, called tiles. The processing of pathological original images included several steps. (1) The raw tiles with 512 × 512 pixels (px, 0.25 µm per px). were cropped from the original whole slide image. For the tissue array, the tissue spot on the scan were manually cropped for each patient and then cropped into number of 512 × 512 px raw tiles. (2) To exclude the area of the slides containing no tissue (white background), the tiles with large background were dropped if background account for more than 20% of the tiles. The input sizes of the networks varied, and they were finally resized to a resolution of 224 × 224 px.

MobileNet V2 training strategy

MobileNet V2 was developed using MIL for training only on tile collections that carried a label for the associated original image. Before entering the network, each tile is distorted and normalized. First, it is randomly cropped to a size of 224x224 px, before the orientation of the tile is distorted. After nuclei segmentation, the color-normalized RGB tiles were then concatenated with their heat map in channel level to produce a 4-channel tile. For data augmentation, the tile is randomly flipped from left to right (around its central vertical axis), then randomly flipped from top to bottom (around its central horizontal axis), and finally randomly rotated by either 0°, 90°, 180° or 270°. Then, these bags of 4-channel tiles were dumped into a MobileNet V2 model, and the score of each tile was calculated. We deployed a Stochastic Gradient descent (SGD) as the optimization algorithm with a decayed learning rate 0.0001 and halves every 10 epochs. Cross-entropy with L2 regulation was selected as the loss function, and regularized factor $\alpha$ was 0.02.
